# Microstructure of unsaturated loess and its influence on strength characteristics

**DOI:** 10.1038/s41598-022-05464-9

**Published:** 2022-01-27

**Authors:** Ya-zhi Wei, Zhi-hua Yao, Xiao-lei Chong, Jian-hua Zhang, Jun Zhang

**Affiliations:** 1 College of Aeronautical Engineering, Air Force Engineering University, Xi’an, 710038 China; 2 China Southwest Geotechnical Investigation & Design Institute Co., Ltd, Chengdu, 610052 China

**Keywords:** Solid Earth sciences, Geology, Structural geology

## Abstract

Previous studies have shown that structure has a significant influence on the mechanical deformation of unsaturated loess, but there is little published information focused on the influence mechanism of microstructure and mesostructure on the mechanical properties of loess. In this paper, the unsaturated undisturbed loess and its remolded loess under the same physical condition were taken as the research objects. The unsaturated triaxial shear tests with constant suction and net confining pressure were carried out, and the microstructure differences between the two are compared by using SEM and CT scanning to reveal the influence of structure on strength characteristics. The test results show that the cohesion and internal friction angle of undisturbed loess are greater than those of remolded loess. The angle of undisturbed soil particles is obvious, and the particles are bracket contact with good cementation. The remolded loess particles are close to round shape, and the particles are inlaid contact with destroyed cementation. The average radius of undisturbed soil is higher than that of remolded soil, indicating that there are bracket pores in undisturbed soil, but the bracket structure and macropores are deformed during shear deformation, and good structural and cementation ensure the strength of loess specimens.

## Introduction

Soil structure refers to the arrangement and connection between soil particles, which is an important mechanical feature of loess, and has a direct impact on the actual engineering mechanical properties^1–9^. With the increase of the number of engineering projects in loess areas, the related research on loess properties is also being deepened, and structure is one of the important parts. The structure of undisturbed soil has an impact on its mechanical properties in the process of shearing until the failure, and its structure gradually decreases after the failure of the undisturbed soil. Besides, the corresponding mechanical properties also change, which can be considered as manifestation of the transition to non-structural remolded soil. Therefore, in-depth study of the structural differences between loess, especially between undisturbed and remolded soil, is helpful to further reveal the mechanical deformation mechanism of loess, and provide theoretical guidance for engineering construction.

Starting with the study of soil microstructure proposed by Terzaghi^10^, he found that honeycomb structure was the most common in clay, and then several models describing microstructure were established. Subsequently, Foldschmidt and Casagrand^11^ proposed two new structures, i.e., the card and book structure of soil, which developed the soil structure on the basis of the original structure. Mitchell^12^ proposed the concept that soil structure is the arrangement of particles and the role of cementation. Leroueil^13^ proposed that soil should be called structural soil. Affected by structure, undisturbed and remolded soil have fundamental differences in mechanical properties. At present, many scholars have studied the structural characteristics and mechanical deformation characteristics of soil from the macro and micro perspectives.

From a macro perspective, HadžiNiković, Gordana D^14^ studied the natural samples of loess sediments with undisturbed macroporous structure and loess sediments with damaged soil structure, and analyzed the influence of natural soil structure on unsaturated shear strength. On this basis, the undisturbed and remolded loess were gradually taken as the research object, combined with the triaxial test, from the perspective of ultimate strength, tensile strength, shear strength and parameters, by comparing the strength differences between undisturbed and remolded loess, the influence of structure on strength was discussed^15–19^. In addition, because unsaturated loess is formed under arid and semi-arid climate conditions, its special sensitivity to water—collapsibility has always been a subject of concern in geotechnical engineering academic and engineering circles. Therefore, collapsibility tests and suction control triaxial tests were carried out on undisturbed and remolded loess specimens, and used the concept of disturbance state (DSC) to characterize the stress–strain behavior of undisturbed and remolded specimens of collapsible loess in the shear process, so as to study the influence of disturbance and structure on hydraulic characteristics of collapsible loess^20,21^. The obvious feature of collapsible loess is that it may produce large additional compression deformation after immersion and it is the basic work for the formulation of compaction standards to know well the characteristics of high pressure compression and additional compression deformation of compacted loess. Therefore, the compression characteristics of undisturbed and compacted loess through a series of suction control measuring instruments were studied, and the influence of structure on the hydraulic properties of loess specimens from a macroscopic point of view was discussed^22^. Based on a series of indoor tests, the influence of structure on the mechanical properties of unsaturated loess through theoretical calculation was quantified^23^. The above researches provide an important research basis for further understanding the influence of structure on the mechanical properties of loess, but the results obtained from the macro perspective cannot fully explain the influence mechanism of micro-structure on the mechanical properties of loess.

Microcosmic method is another perspective for analyzing soil structure, which can be used to more intuitively understand soil particle morphology, connection mode and pore characteristics. Electron microscopy was widely used in the 1950s. In 1959, Rosenqvist applied electron microscope to the study of soil microstructure for the first time^24^. By the 1970s, the use of scanning electron microscope had made a qualitative leap. At present, it is the most common method in the study of soil microstructure. The undisturbed and remolded loess were used as the research object, combined with macroscopic triaxial test, on the basis of studying the difference of shear characteristics between them, scanning electron microscope (SEM) was used to observe the microstructure characteristics of loess specimens before and after triaxial test. The influence of microstructure and structural change on strength and deformation characteristics of loess were analyzed^25–28^. Meanwhile, the structure and mechanical properties of natural collapsible loess and acid treated loess were studied and compared, and analyzed the difference of microstructure by using scanning electron microscope (SEM)^29^. With the development of observation technology, the researchers combined SEM with energy dispersive X-ray (EDX) and X-ray diffraction (XRD) to detect the change process of strength parameters and microstructure of natural loess and alkali-treated loess at four different concentrations, and analyzed the evolution results of shear strength index and stress–strain characteristics of loess^30,31^. In the late 1980s, CT technology began to be applied to soil science and was introduced into the field of geotechnical engineering. With its technical characteristics of non-destructive detection and real-time scanning during the test, it can qualitatively and quantitatively characterize the microstructure parameters of loess and analyze the microstructure evolution in the process of loess collapse^32^. On this basis, CT technology made it possible to study the three-dimensional internal fracture propagation of loess under different test conditions^33^, and proposed a method to quantitatively describe the loess structure and related evolution laws^34^ to reflect the relevant mechanical properties of unsaturated structural loess^35^. The above studies discussed the shear strength of loess from different perspectives through micro-structure. Moreover, to the authors' best knowledge, there is little published information focused on the influence mechanism of microstructure (electron microscope scanning) and mesostructure (CT scanning) on the mechanical properties of loess.

It is known that there is a big difference in strength characteristics between undisturbed loess and remolded loess. Therefore, it is of great significance to analyze the influence of structure on the strength characteristics of loess by combining macroscopic triaxial shear with microscopic structure. In this paper, the macroscopic triaxial tests with controlled suction and confining pressure are carried out on unsaturated undisturbed and remolded loess specimens. The differences of micro-structural characteristics between undisturbed and remolded loess in particle size, skeleton, porosity and other aspects are compared through microscopic and mesoscopic approaches. Based on this, the reasons for the differences in shear strength between undisturbed and remolded loess in macroscopic triaxial tests controlling suction and confining pressure are analyzed, which provides an important experimental basis and scientific reference for further revealing the influence of structure on the strength characteristics of loess. The main structure of this paper is as follows. In the second section, the test scheme (triaxial shear test, electron microscope scanning and CT scanning) and materials are described in detail. In the third section, the stress–strain relationship and strength parameters are mainly described. In the fourth section, the influence mechanism of the differences in the microstructure characteristics, pore structure distribution characteristics and water holding characteristics of undisturbed loess and remolded loess on the strength characteristics of loess is discussed.

## Materials and methods

### Macromechanical test

#### Specimen preparation

**Figure 1:**
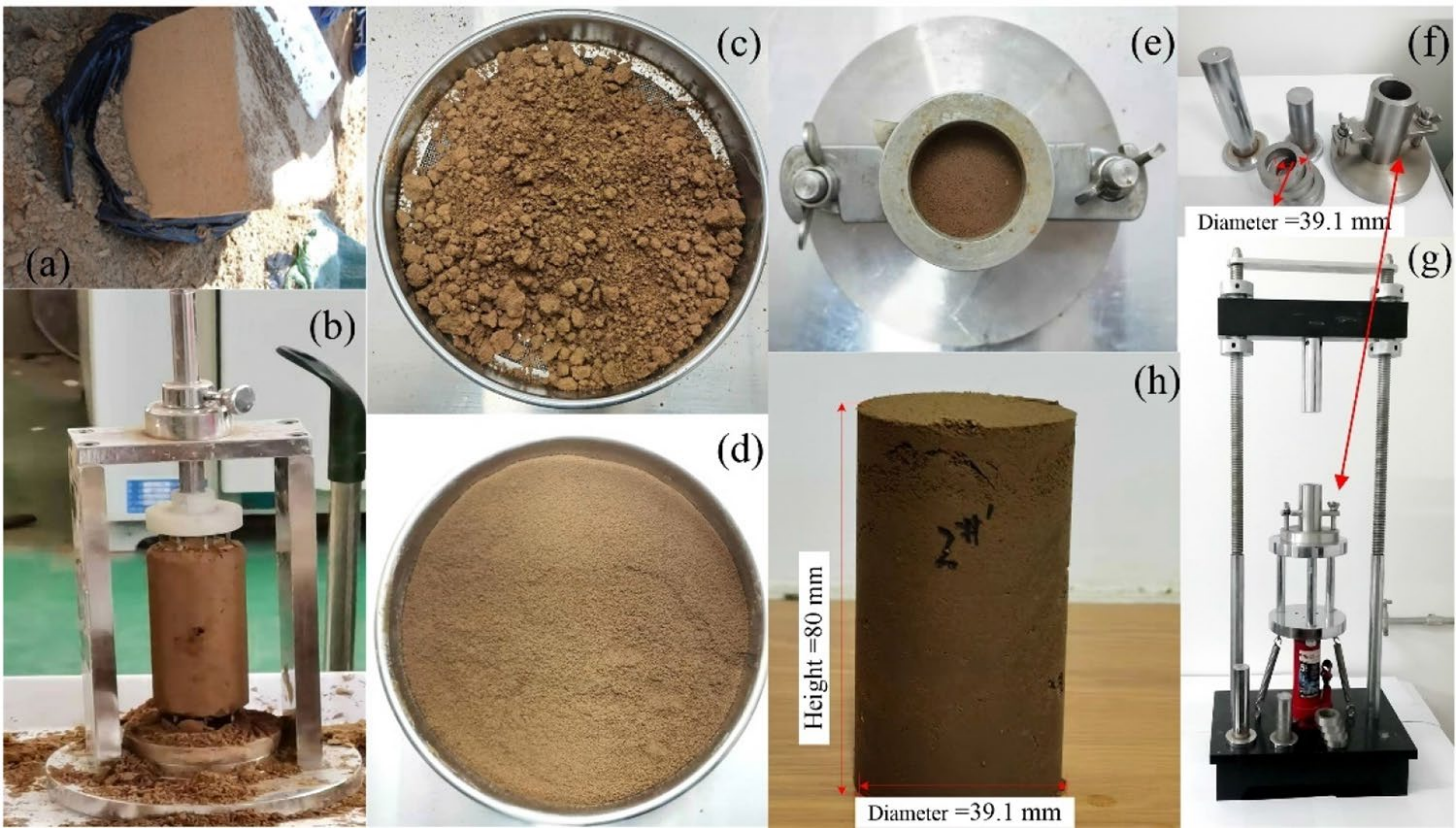
Sampling equipments and methods for undisturbed and remolded loess. (**a**) The undisturbed loess specimen cut on site. (**b**) Undisturbed loess cutter. (**c**) Remolded loess specimen before crushing through the 1 mm sieve. (**d**) Remolded loess specimen after crushing through the 1 mm sieve. (**e**) The remolded loess specimen is compacted by dividing 5 layers. (**f**) Moulds for pressing remolded loess specimen. (**g**) The remolded specimen. (**h**) The remodeling specimen preparation equipment.

The specimens are taken from Weiyang District, Xi’an City, Shaanxi Province. The exploratory wells are manually excavated at the site. Several undisturbed loess specimens (30 × 30 × 30 cm) are obtained under the surface 5 m (as shown in Fig. 1a). To prevent the moisture from evaporation, plastic film is used to wrap the specimens for transporting them back to the laboratory. The relative density of loess particles used in the test is 2.71, and its physical indexes are shown in Table 1.

**Table 1:** Initial physical index of undisturbed loess specimens.

Initial moisture content	Dry density	Void ratio	Saturation	Liquid limit	Plastic limit
*w* (*%*)	$\rho_{d}$ (g/cm^3^)	E	S_*r*_ (%)	w_*L*_ (%)	*w*_P_ (%)
13.45	1.35	1.01	36.09	27.32	16.94

The undisturbed triaxial specimen is made into a cylindrical shape in the diameter of 39.1 mm and the height of 80 mm by using a cutter (as shown in Fig. 1b). The remolded specimen is prepared by using the residual undisturbed loess specimen (as shown in Fig. 1c) which is quickly crushed through the 1 mm sieve (as shown in Fig. 1d). The moisture content and dry density are controlled to be the same as those of the undisturbed loess specimen, and the loess mass is prepared by dividing 5 layers (as shown in Fig. 1e) and the same amount is placed in the device (as shown in Fig. 1f) layer by layer. The specimen is compacted by the remolded loess specimen preparation equipment (as shown in Fig. 1g), and the remolded specimen size is the same as that of the undisturbed loess specimen (as shown in Fig. 1h).

The initial moisture content of the specimen is 13.45%. According to previous experience, if the matrix suction should be controlled, the moisture content of the specimen has to be increased. In this paper, the water film transfer method is used to increase the moisture content of the specimen to 20%. The water is evenly dripped on the undisturbed specimen for several times at 2–3 h intervals. The specimens after the adjustment of the moisture content are placed in the humidifier, and rotated once every 12 h. The weight change of the specimens is weighed to ensure that the target moisture content is achieved. The specimen is placed in a humidifier for not less than 72 h. The initial moisture content of remolded loess specimens is the same as that of undisturbed loess specimens, which is 13.45%, and the moisture content is increased to 20% by using water film transfer method.

#### Test steps

**Figure 2:**
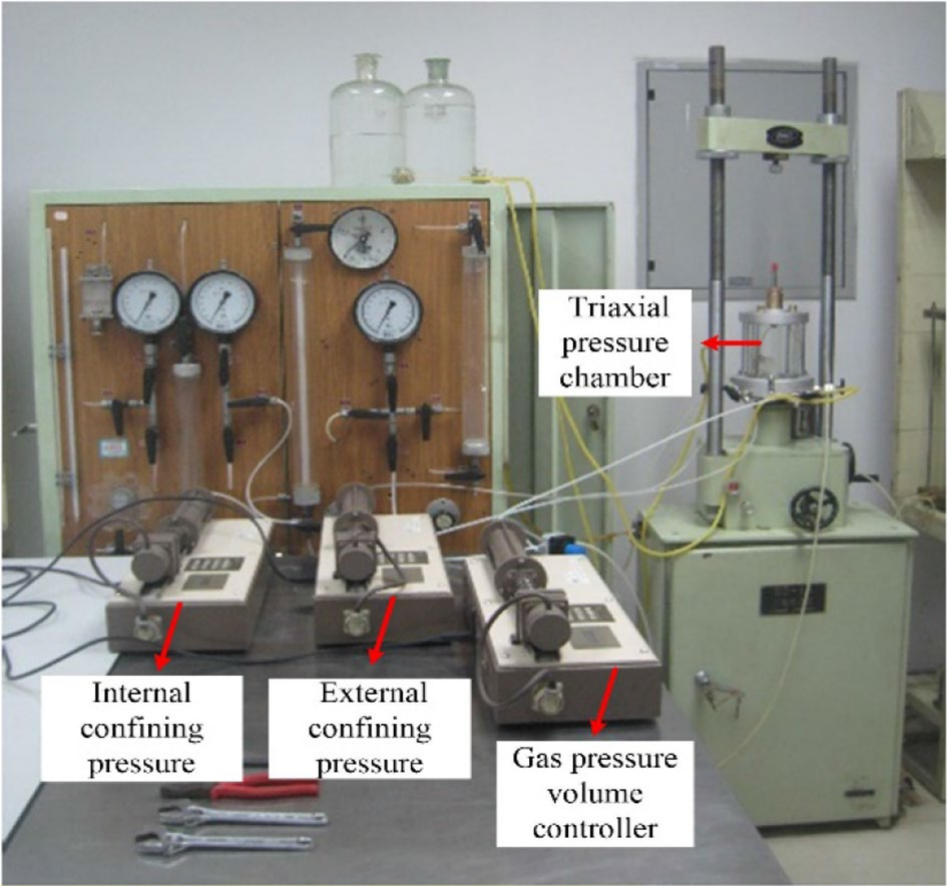
Unsaturated triaxial apparatus.

In this paper, the triaxial consolidation draining shear test with constant suction and net chamber pressure is adopted. The triaxial apparatus of unsaturated loess is shown in Fig. 2. The pressure chamber used in this test is a double-layer pressure chamber, and two pressure volume controllers are used to control the confining pressure (internal pressure and external pressure) respectively, in order to reduce the deformation of the pressure chamber and avoid the error caused by the volume change test of the sample. In the test, the confining pressure is controlled by three levels, namely 50, 100 and 200 kPa, and the initial suction is controlled at 50, 100, and 200 kPa, respectively, and they are applied by the gas pressure volume controller. There are 9 undisturbed specimens and remolded specimens each, i.e., 18 specimens in total. The specimen suction balance and consolidation process stability standard is within 2 h volume change, and displacement does not exceed 0.01 cm^3^. Besides, the volume change is tested by using GDS pressure volume controller. Triaxial shear rate is 0.016 mm/min, if the stress–strain curve has a peak, the peak of the principal stress difference on the curve is taken as the failure point. In the case that there is no peak, when the axial strain reaches 15%, take the corresponding principal stress difference as the failure point, and stop shearing.

### Microscopic and mesoscopic scanning

#### Microscopic scanning specimens and apparatus

To reveal the difference of macroscopic mechanical behavior between undisturbed and remolded loess, the microstructure of the two kinds of loess specimens is analyzed by scanning electron microscope. Before SEM scanning, the prepared triaxial undisturbed and remolded loess specimens are cut into strips of about 2 × 1.5 × 1.5 cm with a small knife, and a circle of grooves with the depth of about 2 mm is carved in the middle. Then, the grooves are broken down, and a relatively flat section is selected, and the dust is blown off with a rubber ball. After that, the specimens are adhered to the base of the observation room, and the VEGA II XMU scanning electron microscope is used for scanning. The resolution is 40.2 μm. During the scanning process, the specimen is moved into the field of view, and the representative areas are selected to take pictures, thus avoiding the singular points. Each area is photographed from high magnification to low magnification, mainly including 50, 500, 5000 and 10,000.

#### Mesoscopic scanning specimens and apparatus

For the CT three-dimensional structure reconstruction scanning, the undisturbed and remolded ring-knife specimens are prepared first, with the diameter of about 61.8 mm and the height of about 20 mm. To avoid the influence of metal on CT scanning, the ring cutter is made of Peek material (polyetheretherketone) with high strength and processability into the parts in precise size. The apparatus used in this specimen test is the industrial CT with high spatial resolution of nanoVoxel-2000 series equipped with 3 μm (flat panel detector)/500 nm (objective coupling detector). Using this apparatus for data acquisition, and based on CT data results, quantitative analysis of the specimen internal porosity and connectivity, statistical pore radius data is implemented. High-resolution scanning imaging of the specimen is conducted, an image is taken every 0.25° in the size of 1920 × 1536. To improve the resolution, the specimen rotates for two circles, and a total of 1440 frame images are taken. Other test parameters are shown in Table 2. The three-dimensional reconstruction is carried out according to the CT scanning results of loess specimens, and then the pores are extracted combined with the three-dimensional image to analyze the differences in the pore structure of undisturbed and remolded loess.

**Table 2:** CT scan related test parameters.

Resolution ratio/μm	Voltage/kV	Electric current/μA
40.20	180	120
Time of exposure/s	Scanning time/min	
0.60	70	

## Test result and analysis.

### The stress–strain relationship between undisturbed and remolded loess

**Figure 3:**
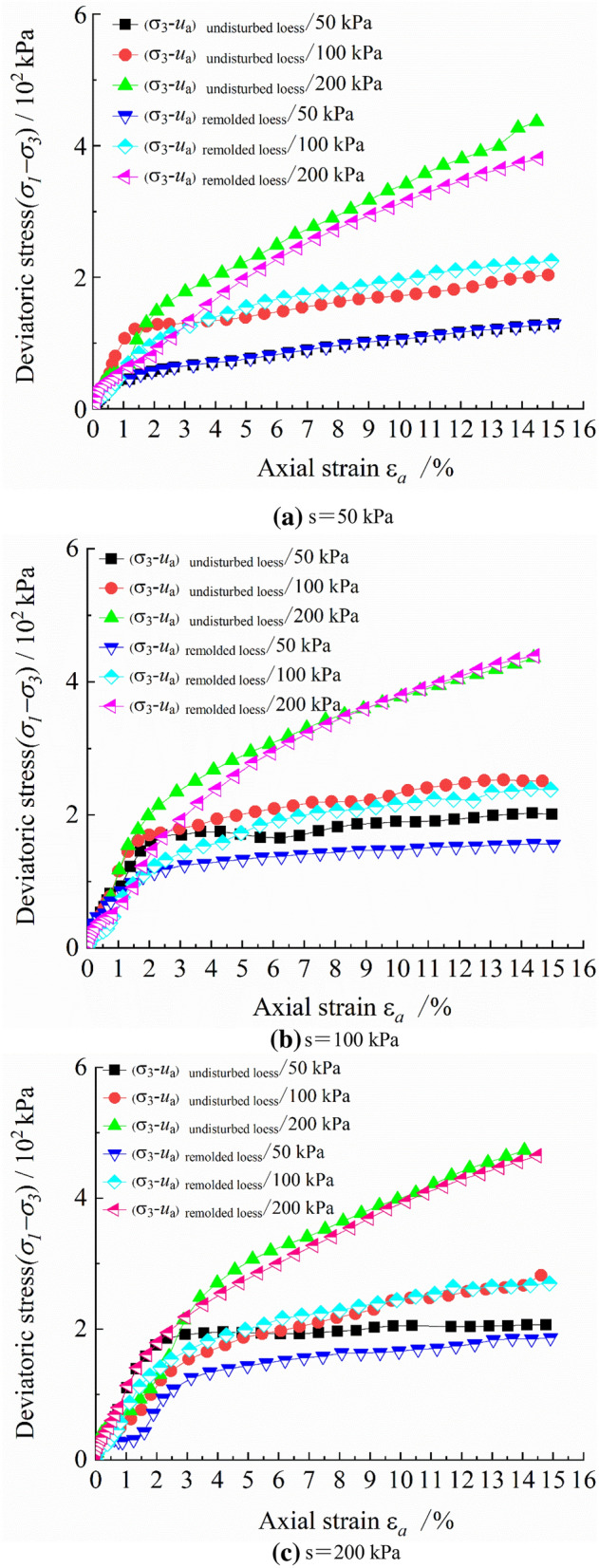
Curves of deviatoric stress and axial strain of unsaturated undisturbed and remolded loess under the same suction and different net confining pressure.

**Figure 4:**
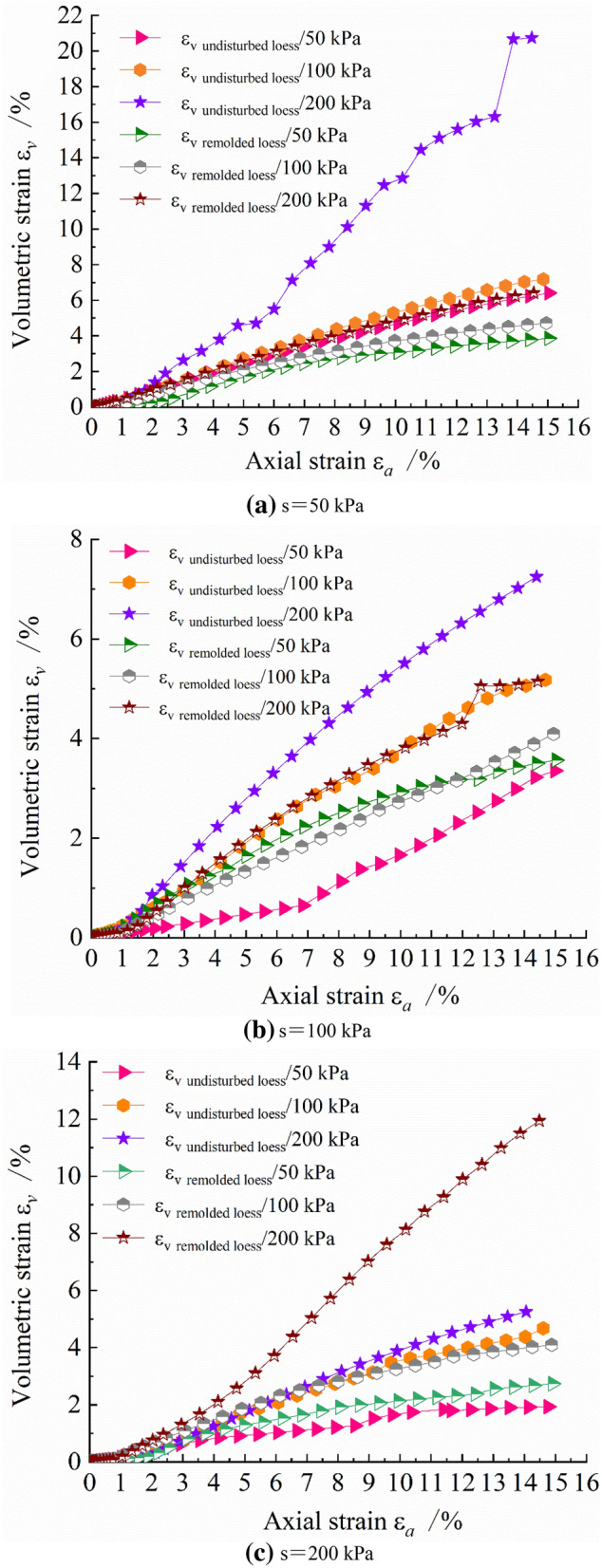
Curves of volume strain and axial strain of unsaturated undisturbed and remolded loess under the same suction and different net confining pressure.

Figure 3 is the $\left( {\sigma_{1} { - }\sigma_{3} } \right){ - }\varepsilon_{a}$ relationship curve and Fig. 4 is the $\varepsilon_{v} { - }\varepsilon_{a}$ relationship curve of triaxial draining shear tests of 18 specimens under different net confining pressures and the same suction condition. It can be seen from the figure that when the matric suction is the same, the deviatoric stress of undisturbed and remolded loess specimens increases with the increase of net confining pressure of $\left( {\sigma_{3} { - }u_{a} } \right)$, and when the axial strain is constant, the deviatoric stress of undisturbed loess is slightly higher than that of remolded loess. The deviatoric stress–strain curve of undisturbed loess develops from weak hardening to strong hardening. The deviatoric stress–strain curve of remolded loess at low confining pressure tends to be softening curve, and it shows hardening curve at high confining pressure. However, the shear strength of both loess increases with the increase of suction. When the axial strain $\varepsilon_{a}$ is small, the slope of the initial curve of deviatoric stress $\left( {\sigma_{1} { - }\sigma_{3} } \right)$ of undisturbed loess specimen is obviously larger. At the same time, it can be seen that the smaller the net confining pressure, the steeper the initial curve of deviatoric stress $\left( {\sigma_{1} { - }\sigma_{3} } \right)$. At relatively large $\varepsilon_{a}$, when the deviatoric stress increases gradually, the slope of the curve first decreases gradually and then stabilizes. The reason is that the lower confining pressure is not enough to damage the structure of loess at the early stage of shear. For remolded loess specimens, no matter whether the confining pressure is large or not, there is a cross phenomenon in the stress–strain diagram, indicating that the strength failure of loess at the initial stage of deviatoric stress is obvious. In addition, when the axial strain is small, the slope of the curve is large. With the increase of axial strain, the slope of the curve is basically stable. The deviatoric stress of intact loess increases rapidly at the axial strain of about 1—1.5% ; while when the axial strain of remolded loess reaches 3% , the deviatoric stress increases to a larger value.

Under different confining pressures, the volumetric strain $\varepsilon_{v}$ of undisturbed loess increases gradually with the increase of axial strain $\varepsilon_{a}$. When the net confining pressure is large, the structure between loess particles is destroyed, and the internal microstructure cannot withstand external load, resulting in the increase of loess specimen deformation. In addition, when the axial strain ratio is small, the slope of the volumetric strain – axial strain curve is large. As the axial strain increases gradually, the slope of the curve decreases gradually, indicating that at the initial stage, the volumetric deformation rate of the loess specimen is fast. When a certain deformation is reached, the volumetric deformation rate decreases gradually, and the volumetric deformation difference decreases. The change trend of volumetric strain of remolded loess specimen with axial strain is the same. The main difference is that when the suction is large, the volumetric strain of remolded loess specimen is larger than that of undisturbed loess specimen under the same conditions. The remolded loess specimen is formed by the crushing and reorganization of the undisturbed loess specimen. Even if the particles are rearranged to form a new granular structure, the connection between particles has been destroyed, therefore, the structural strength and structural stability are not as good as those of the undisturbed loess specimen.

### Strength parameters analysis of undisturbed and remolded loess

The $\left( {\sigma_{1} { - }\sigma_{3} } \right){ - }\varepsilon_{a}$ curve of triaxial drained shear test with constant suction and net confining pressure is shown in Fig. 4. It can be seen from the figure that the triaxial shear test of unsaturated undisturbed and remolded loess is hardened and broken, and the stress of axial strain $\varepsilon_{a} = 15{\text{\% }}$ is taken as the breaking stress. The deviatoric stress $q_{f}$ and net mean stress $p_{f}$ of the specimen at failure can be calculated by the following formulas:

1 $$q_{f} = \frac{{\left( {\sigma_{1} - \sigma_{3} } \right)_{f} }}{2}$$

2 $$p_{f} = \left[ {\frac{{\left( {\sigma_{1} + \sigma_{3} } \right)}}{2} - u} \right]_{f}$$

where $\sigma_{1}$ refers to the major principal stress (kPa), $\sigma_{3}$ represents the minor principal stress of the specimen, which is equal to the confining pressure (kPa) applied in the test, $\left( {\sigma_{1} { - }\sigma_{3} } \right)_{f}$ refers to the major principal stress difference (kPa) when the specimen is broken, $u$ represents the suction.

The deviatoric stress $q_{f}$ and net mean stress $p_{f}$ of undisturbed and remolded loess specimens are listed in Table 3.

**Table 3:** $q_{f}$ and $p_{f}$ of unsaturated loess under triaxial shear failure.

Suction *u*	Net confining pressure	$q_{f}$(*kPa*)		$p_{f}$(*kPa*)	
(*kPa*)	(*kPa*)	The undisturbed loess	The remolded loess	The undisturbed loess	The remolded loess
50	50	62.33	54.40	62.33	54.40
	100	109.41	84.51	159.41	134.51
	200	172.29	163.82	322.29	313.82
100	50	92.62	80.12	42.62	30.12
	100	139.14	127.23	139.14	127.23
	200	218.05	205.33	318.05	305.33
200	50	113.71	101.68	48.33	36.29
	100	173.92	152.91	103.92	92.91
	200	250.79	234.10	250.79	234.10

**Figure 5:**
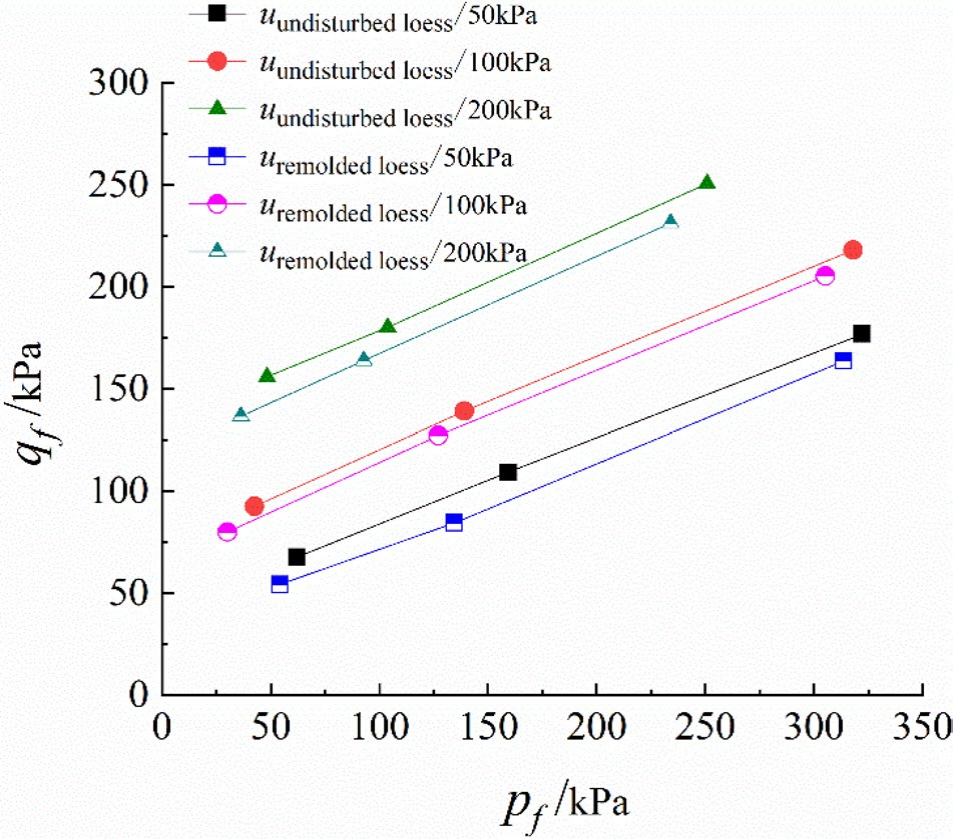
Strength criterion of unsaturated loess in plane.

The relationship between $q_{f}$ and $p_{f}$ is illustrated in Fig. 5 to form the strength envelope in the plane $p_{f} - q_{f}$. It can be seen from this figure that a group of test points with the same suction fall on a straight line, which can be expressed as follows:

3 $$q_{f} = d + p_{f} \,tan\,\psi^{\prime }$$

where $d$ and $tan\psi ^{\prime}$ are the intercept (kPa) and slope of the straight line, respectively. Their values are determined by using the least square method and listed in Table 4.

**Table 4:** Unsaturated loess triaxial shear test parameters.

Suction *u*	Net confining pressure	The undisturbed loess					The remolded loess		
(*kPa*)	(*kPa*)	$d$(*kPa*)	$tan\psi^{\prime }$	$c$(*kPa*)	$\phi^{\prime }$(°)	$d$(*kPa*)	$tan \, \psi ^{\prime}$	$c$(*kPa*)	$\phi ^{\prime}$(°)
50	50								
	100	42.10	0.45	36.37	26.83	29.65	0.40	21.95	23.58
	200								
100	50								
	100	74.37	0.46	43.75	27.39	67.71	0.45	32.99	26.47
	200								
200	50								
	100	132.51	0.47	57.74	28.03	119.35	0.46	50.06	27.39
	200								

According to Mohr–Coulomb condition, pressure is positive and pull is negative. The cohesion $c$ and internal friction $\phi^{\prime }$ angle of soil can be obtained by formula (4):

4 $$\left\{ {\begin{array}{*{20}l} {c = \frac{{q_{f} }}{{cos \,\phi^{\prime } }} - p_{f} tan\, \phi^{\prime } } \hfill \\ {tan\,\psi^{\prime } = sin \,\phi^{\prime } } \hfill \\ \end{array} } \right.$$

**Figure 6:**
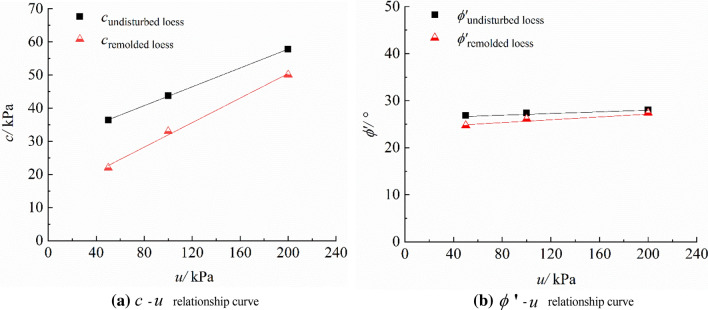
$c - u$, $\phi^{\prime } - u$ relationship curves of unsaturated loess.

The strength parameter $c$ and $\phi ^{\prime}$ can be calculated from the above two formulas. The values are listed in Table 4, and the relationships $c - u$ and $\phi^{\prime } - u$ are shown in Fig. 6a,b, respectively.

It can be seen from Fig. 5 that under different suctions, the distribution range $p_{f} - q_{f}$ of test points is very narrow, and the critical state can be grouped into a straight line, moreover, the critical state line approximately moves down in parallel with the decrease of suction. It can be seen from Fig. 6 that the cohesion of unsaturated undisturbed and remolded loess increases linearly with the suction, and the internal friction angle is less affected by the suction. It can be seen that the cohesion and internal friction angle of undisturbed loess are greater than those of remolded loess, and the difference of internal friction angle is smaller than that of cohesion.

The theoretical Formula (5) of shear strength of unsaturated loess proposed by Fredlund ^36^ et al. is used for the description.

5 $$\tau_{f} = c^{\prime } + (\sigma_{f} - u_{a} )_{f}\,tan\, \phi^{\prime } + (u_{a} - u_{w} )_{f}\,tan\, \phi^{b}$$

where $c^{\prime}$ refers to intercept of stress point envelope on $q$ axis when $r_{f}$ and $p_{f}$ are zero; $r_{f}$ refers to matrix suction at failure; $(\sigma - u_{a} )$ represents the net normal stress on the failure surface; $(u_{a} - u_{w} )$ denotes the matrix suction; $\phi^{b}$ is the suction friction angle, $tan \, \phi^{b}$ is the increasing rate of shear strength with $(u_{a} - u_{w} )$, that is, the contribution rate of matrix suction to the shear strength of unsaturated loess.

The strength parameters of undisturbed and remolded loess specimens are shown in Table 5.

**Table 5:** Unsaturated loess strength parameter list.

Cohesion $c^{\prime}$(*kPa*)		Internal friction angle $\phi ^{\prime}$(°)		Suction friction angle $\phi^{b}$(°)	
The undisturbed loess	The remolded loess	The undisturbed loess	The remolded loess	The undisturbed loess	The remolded loess
23.44	17.12	26.32	23.48	13.95	12.30

The structure of undisturbed loess is relatively stable, which can better resist external damage. The particle connection of remolded loess specimen is not stable, and the resistance is poor. Therefore, the shear strength parameters of undisturbed loess specimen are higher than those of remolded loess. Thus, under the same test conditions, the differences between undisturbed and remolded loess strength indexes are mainly reflected by structural differences.

## Discussion

From the analysis of the results of the previous triaxial shear test, it can be seen that the structure of the undisturbed loess is relatively stable, which can better resist the damage of the external force, and the particle connection of the remolded loess specimen is unstable with poor resistance. It can be explained that under the same test conditions, the structural differences between the undisturbed and remolded loess lead to differences in their strength indicators. Therefore, it is necessary to conduct in-depth research on the microstructure characteristics of the undisturbed and remolded loess, and further analyze the influence mechanism of the structure on the strength of the loess.

### Impact mechanism of microstructure characteristics on strength of unsaturated loess

**Figure 7:**
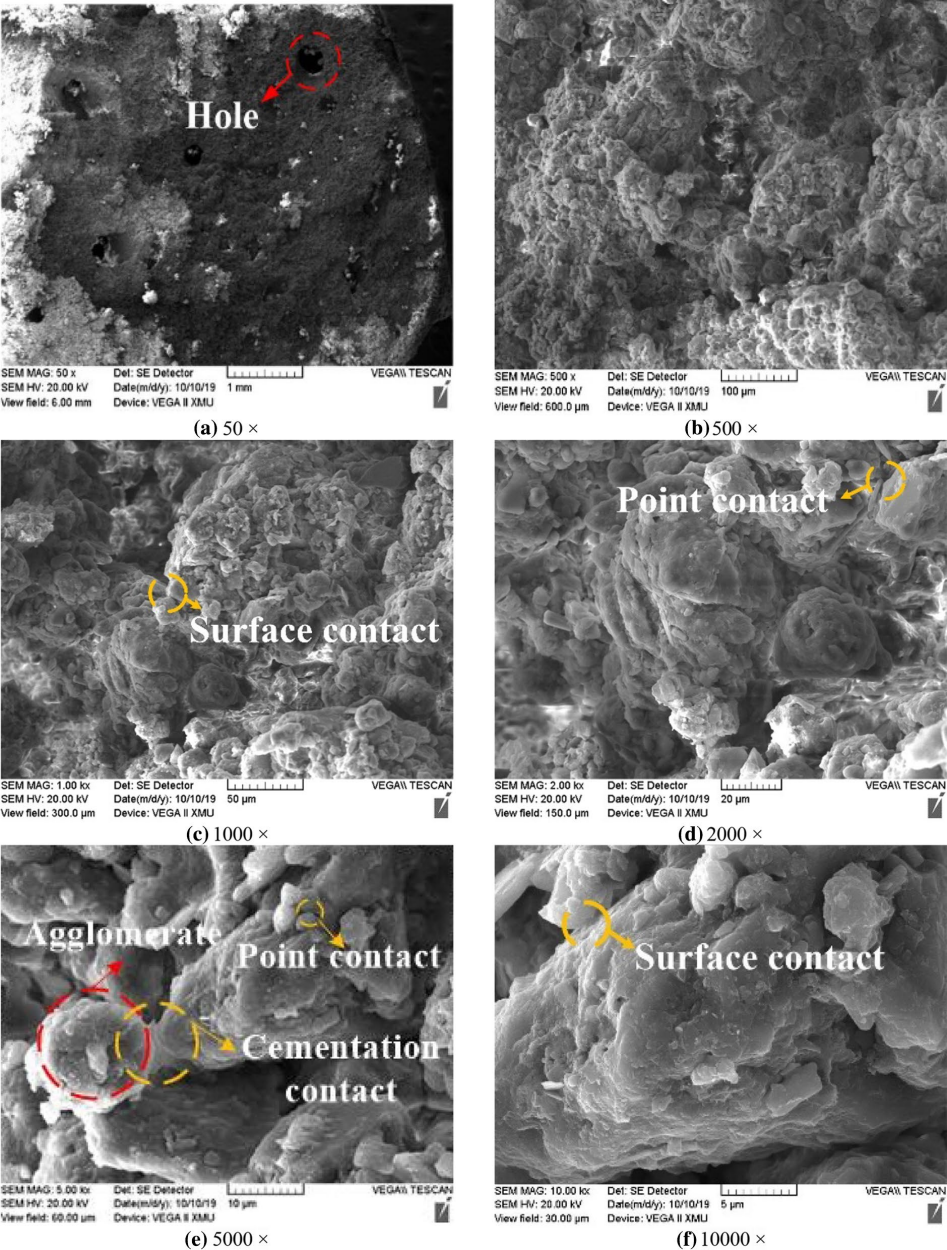
SEM image of undisturbed loess.

**Figure 8:**
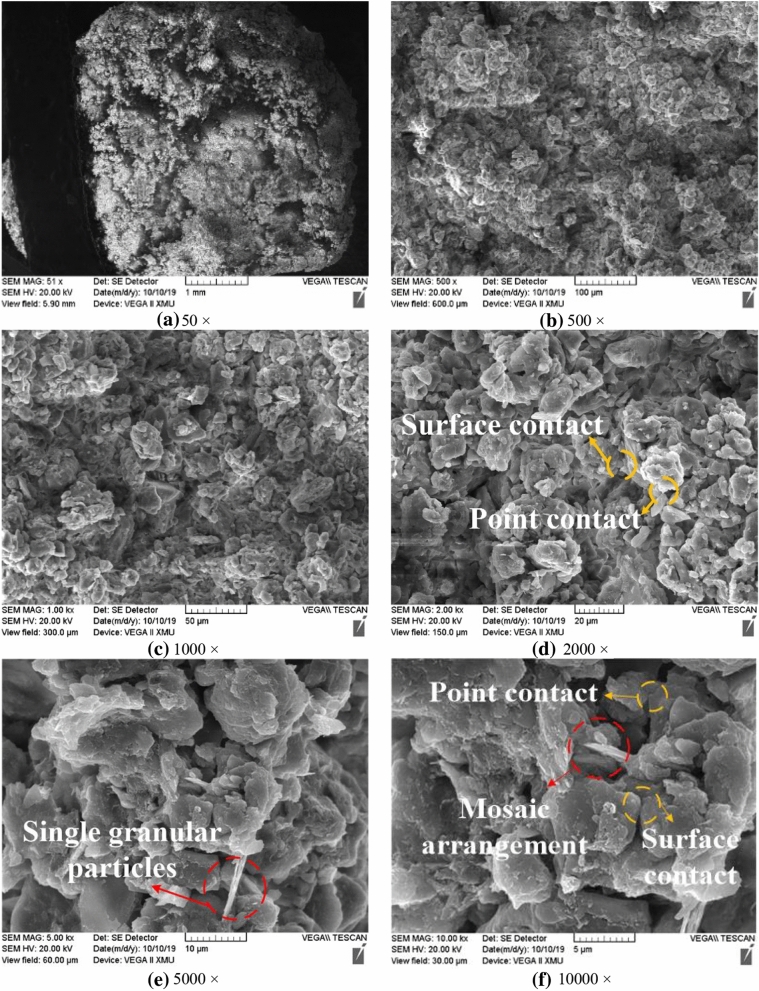
SEM image of remolded loess.

The structural scales of loess specimens observed under different magnifications during SEM scanning are also different. This paper lists the SEM images of undisturbed and remolded loess specimens with magnifications of 50–10,000, as shown in Figs. 7 and 8^37^ . When 50 is enlarged, the undisturbed loess shows obvious large holes (Fig. 7a), while the remolded loess does not exhibit this feature (Fig. 8a). When the magnification is 500–5000 (Figs. 7b,c, 8b,c), very clear morphology of loess particles can be observed, in which the skeleton particles of undisturbed loess are mainly aggregates cemented by fine particles, and the particles have obvious corners; the microstructure of remolded loess is destroyed due to disturbance, therefore, the aggregate of skeleton particles is less than that of undisturbed loess, there are more single particles, and the angular shape of particles is close to round shape. When the magnification is 10,000 (Fig. 7d, 8d), the arrangement form and contact relationship of loess particles can be observed. The skeleton particles of undisturbed loess are mainly arranged in bracket, and the contact relationship is mainly point contact, surface contact and cementation contact. The skeleton particle arrangement of remolded loess is mainly mosaic arrangement, and the contact relationship is mostly point contact and surface contact.

From a macro perspective, the structure of loess shows the deformation of loess in the process of stress and the stress and strain after loess failure. From the corresponding micro perspective, it is manifested as the shape of loess particles, the movement and change of particles in the process of stress, the bonding effect between particles, and the contact force^38–41^. First of all, from the perspective of particle shape, the angle of undisturbed loess particles is obvious, the support contact between particles provides the loess specimen with good stability, and the cement between particles provides strong cementation between particles, so that the specimen skeleton can withstand large ultimate destructive force in the triaxial shear process. After artificial disturbance and remodeling, the particle morphology of remolded loess shows no obvious edges and corners close to the muddy circle, which makes the skeleton stability poor and the shear strength weaker than that of the undisturbed loess. Moreover, most of the original cementation contacts are destroyed due to the disturbance process. The strength of the remolded cementation contact decreases greatly, and the loess skeleton effect is not obvious in the macro shear process.

Secondly, it can be seen from the movement of particles in the process of stress, the axial load is applied from the top and bottom in the triaxial shear test. Therefore, the closer the loess particles to the top and bottom, the greater the displacement, the farther the distance from the top and bottom, and the smaller the displacement. When the strain of the specimen is small, the displacement between the adjacent particles on the same horizontal plane is not large. With the gradual increase of strain, the particles near the top and bottom of the specimen will gradually move to the middle of the specimen. At this time, the displacement between the particles will gradually become obvious, and the movement direction of the particles will change. Under the same stress, the particles of the undisturbed loess specimen are oriented earlier than the remolded loess specimen.

Thirdly, from the perspective of the bonding effect between particles, the particles with obvious edges and corners of the undisturbed loess enhance the occlusion and friction between the particles, and the arrangement between the particles also increases, thus further improving the bonding effect between the particles. The degree of its role, the bonding force increases, and the shear capacity of the undisturbed loess specimen is enhanced; after the remolded loess specimen is disturbed by human beings, both the tangential and normal bonding are destroyed, therefore, the number of destruction is much higher than that of the undisturbed loess specimen, the bonding effect is reduced, and the shear resistance is weakened.

Fourthly, from the point of contact force between particles, “force chain” can intuitively describe the size and change of contact force between particles. Inter-particle contact force increases, and interlocking and friction are enhanced, making the “force chain” stronger^42^; the denser the distribution of “force chain” indicates more contact points between particles, therefore, the strength and distribution of “force chain” can reflect the degree of particle arrangement. In general, the number of weak “force chains” in loess specimens is too large, and they will be destroyed under small shear force. While the number of strong “force chains” is less, and they support the load of loess specimens on a relatively stable basis^43^. In the triaxial shear process, when the strain of loess specimen is in the initial stage, the distribution of “force chain” is relatively dense, and the contact force between particles is small. With the increase of strain, the strength of part of the “force chain” in the loess specimen increases, the arrangement between particles increases, and the number of weak “force chain” decreases gradually. The strong “force chain” transfers from the top of the specimen to the bottom in the form of contact force between particles, forming a complete stress structure. The increase of occlusion and friction between particles is sufficient to offset the weakening arrangement caused by the decrease of weak force chain. Therefore, the loess specimen shows strain hardening effect. Since the distribution of strong and weak “force chains” inside the undisturbed loess specimen is denser than that of the remolded loess specimen, the arrangement between particles is stronger. With the increase of strain, the “strong” force chains running through the loess specimen constitute a more stable force skeleton^44^. In terms of macroscopic mechanical properties, the shear strength is higher than that of the remolded loess specimen.

### Impact mechanism of pore structure and distribution characteristics on strength of unsaturated loess

**Figure 9:**
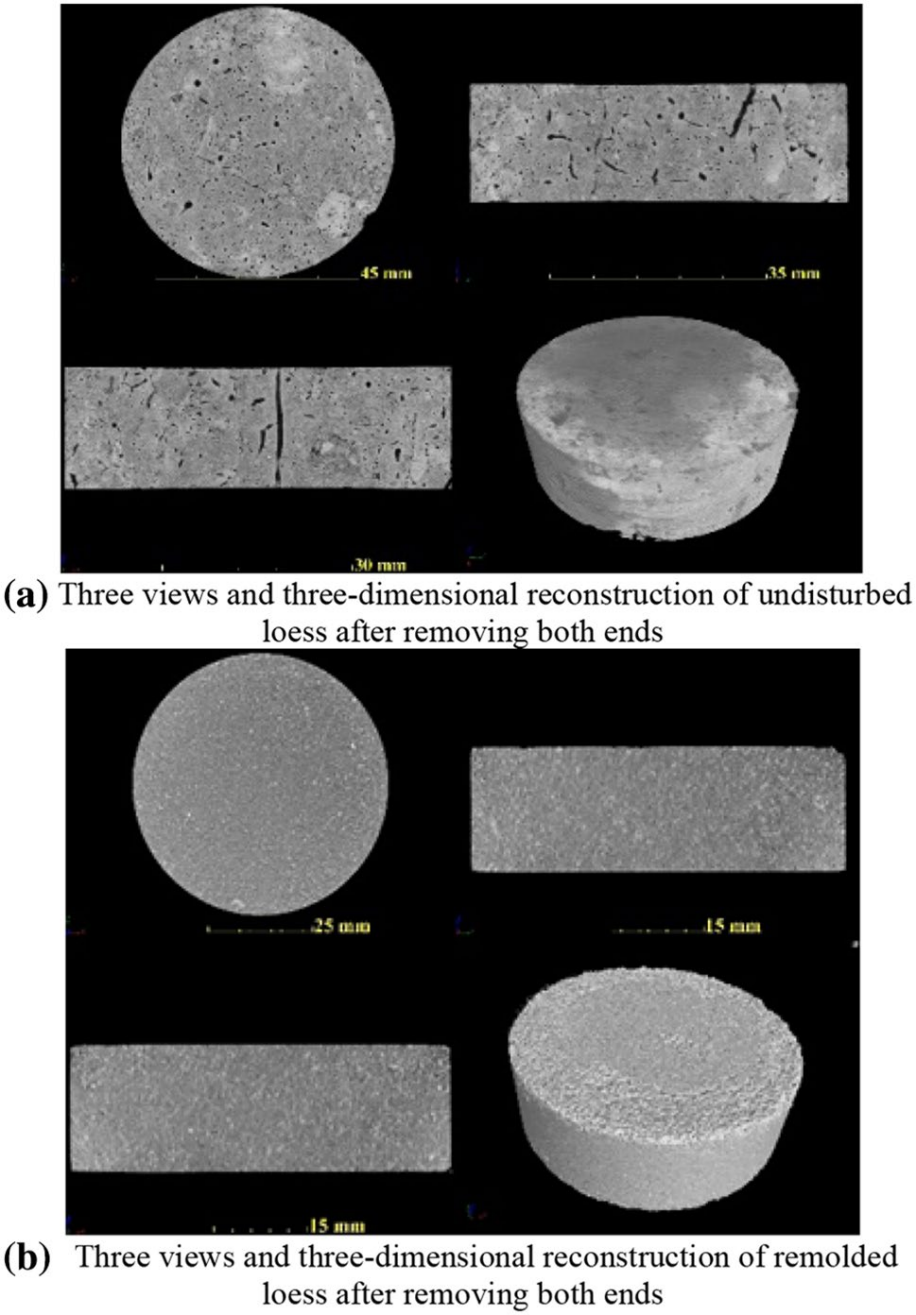
Three-dimensional reconstruction of undisturbed and remolded loess specimen by CT scanning.

**Figure 10:**
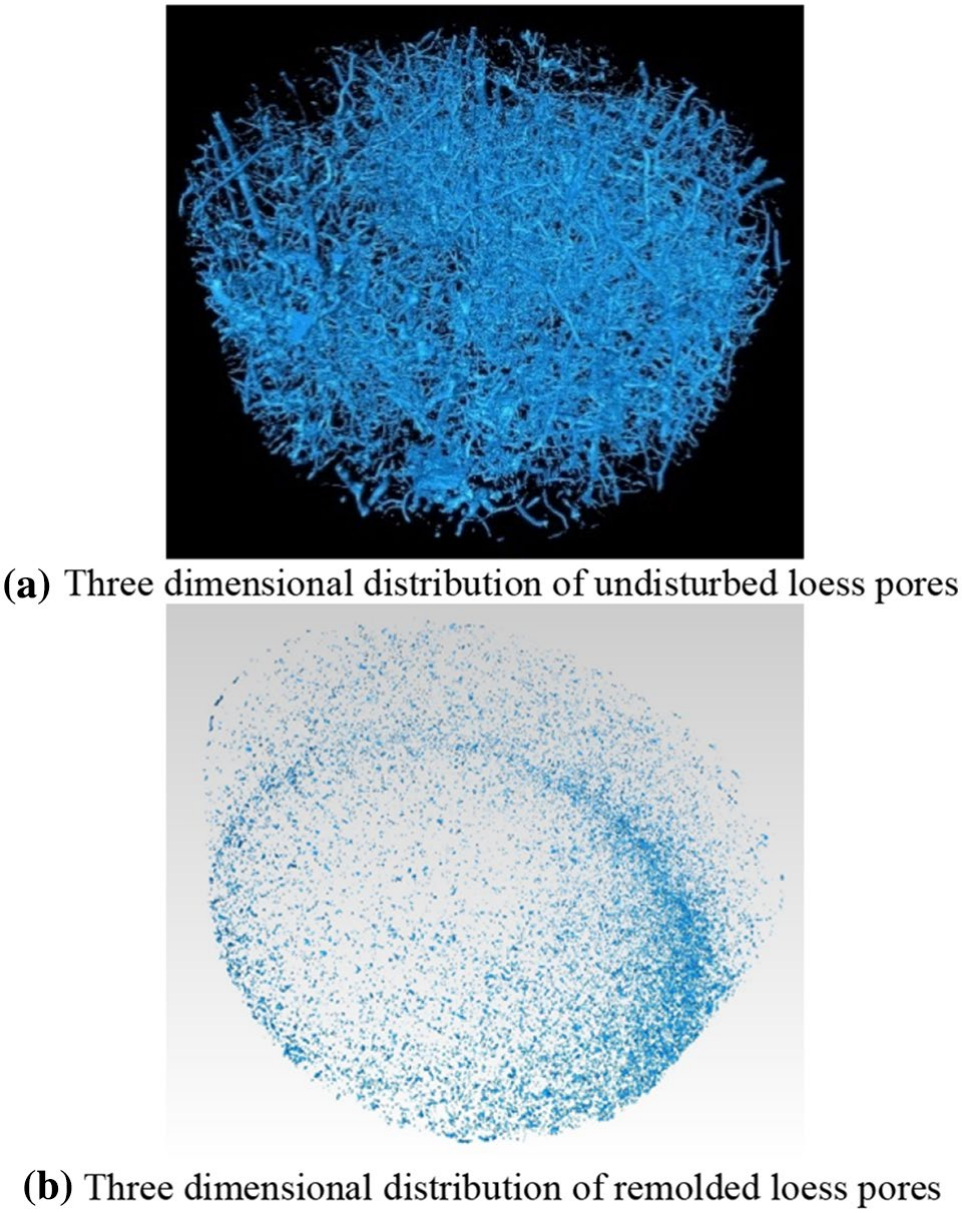
Three-dimensional distribution of pores in undisturbed and remolded loess ring cutting specimen.

**Figure 11:**
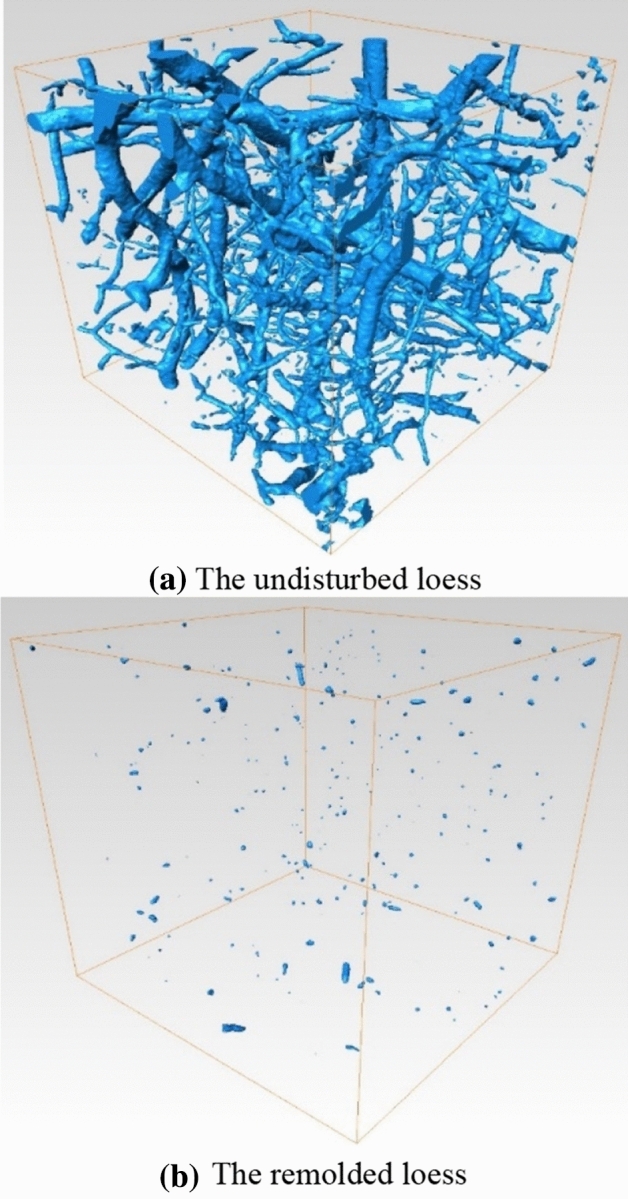
Three-dimensional diagram distribution of specimen interception data pores.

The intact undisturbed and remolded loess specimens are scanned by CT, and three-dimensional reconstruction is carried out. The three-view (top-view, left-view and front view) and three-dimensional reconstruction of the specimen are shown in Fig. 9. It can be seen that the undisturbed loess shows obvious cracks and pores, and the number of large pores is relatively large. The remolded loess specimen is relatively dense without obvious cracks and large pores. From Fig. 10, it is seen more intuitively that the undisturbed loess pores are dense with large pores, while the remolded loess pores are mainly small pores. To further compare the meso-structure of undisturbed and remolded loess specimens, the cube CT data of 300 × 300 × 300 × layer ( side length = 300 × 40.2 μm) are intercepted from the CT scan results of the specimens, and the three-dimensional images of the pores are extracted, as shown in Fig. 11. It can be seen that the pores of undisturbed loess specimens are mostly connected pores, and the pore radius is large. The remolded loess specimen has no connected pores, and the pore radius is generally small, which is related to the connection mode of skeleton particles.

**Figure 12:**
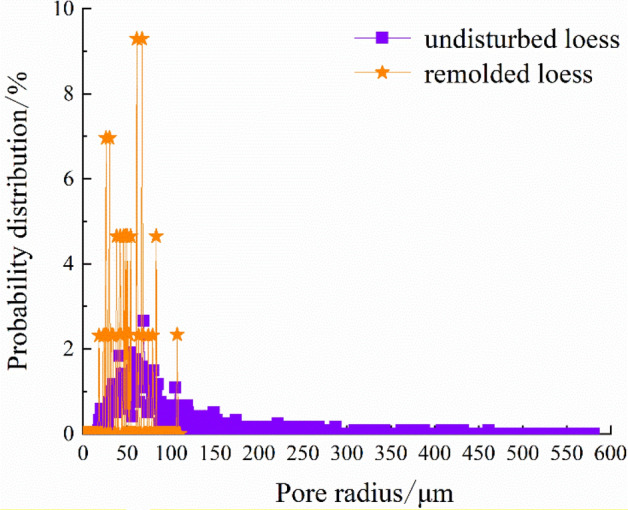
Probability distribution diagram of specimen pore radius.

**Figure 13:**
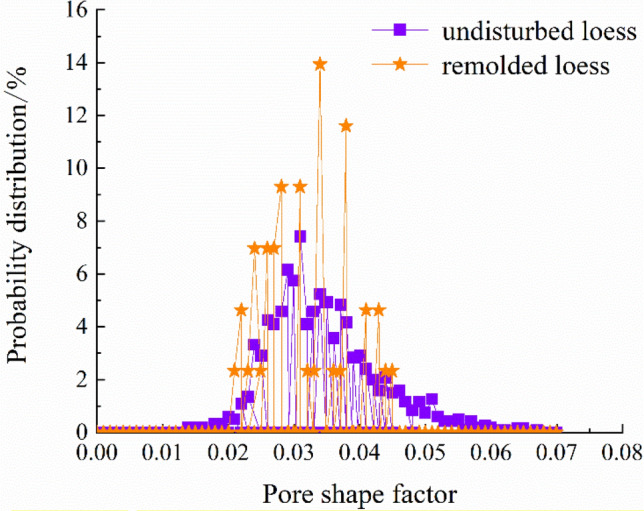
Probability distribution diagram of specimen pore shape factor.

**Figure 14:**
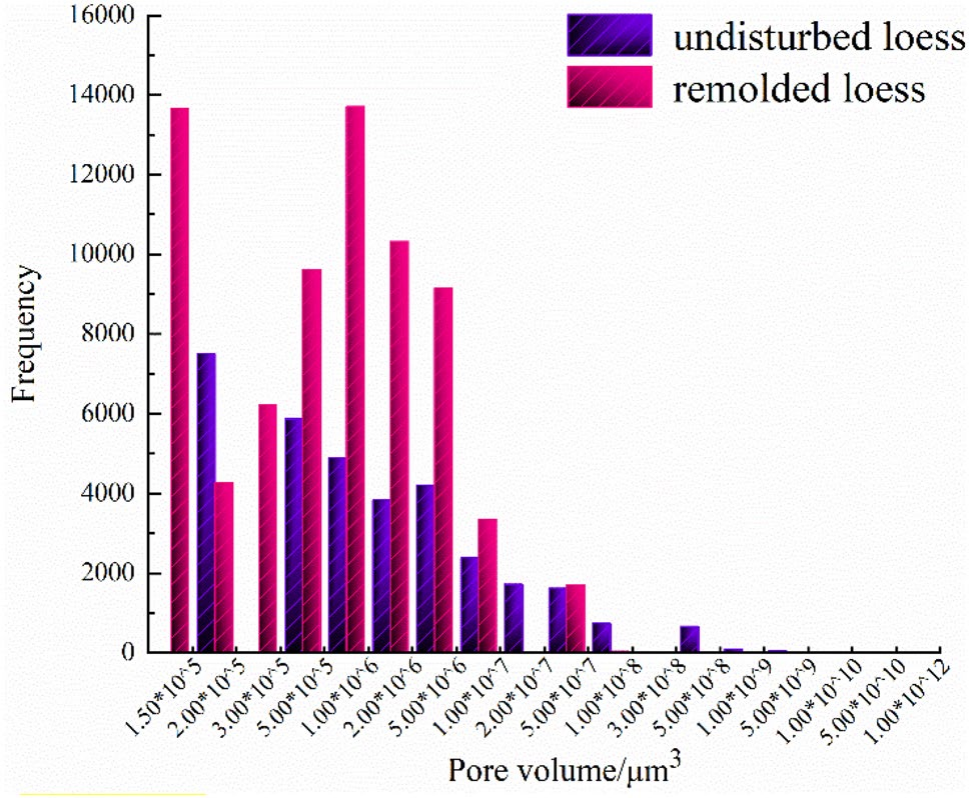
Statistical distribution histogram of specimen pore volume.

The quantitative extraction of pore structure characterization parameters, such as pore radius, pore volume and shape factor (representing the regularity of pore shape, and the larger the value of shape factor, the more regular its shape) can be realized by using mathematical statistics method, as shown in Table 6. And the probability distribution diagram is shown in Figs. 12, 13 and 14.

**Table 6:** Quantitative evaluation of pore structure.

Specimen type	Maximum pore radius/μm	Maximum pore volume/μm^3^	Average pore radius/μm	Average pore volume/μm^3^
The undisturbed loess	460.90	2.44 × 10^9^	88.97	5.33 × 10^7^
The remolded loess	107.02	2.88 × 10^7^	49.95	6.52 × 10^6^

It can be seen from Fig. 12 that the probability distribution of pore radius of undisturbed and remolded loess specimens is skewed distribution, and the pore radius of undisturbed loess specimens is mainly distributed within the range of 0~100 μm, while that of remolded loess specimens is mainly distributed within the range of 15~85 μm. From Fig. 13, the pore shape factor distribution of undisturbed and remolded loess specimens is in normal distribution. The pore shape factor distribution of undisturbed loess specimens is mainly concentrated in 0.024~0.050, and that of remolded loess specimens is 0.022~0.044. The degree of pore shape regularity of undisturbed loess specimens and remolded loess is relatively similar; it can be seen from Fig. 14 that the pore volume of undisturbed loess specimens is mainly within the range of 2.0 × 10^5^ μm^3^~5.0 × 10^6^ μm^3^, and that of remolded loess specimens is mainly within the range of 1.0 × 10^5^ μm^3^~5.0 × 10^6^ μm^3^.

**Figure 15:**
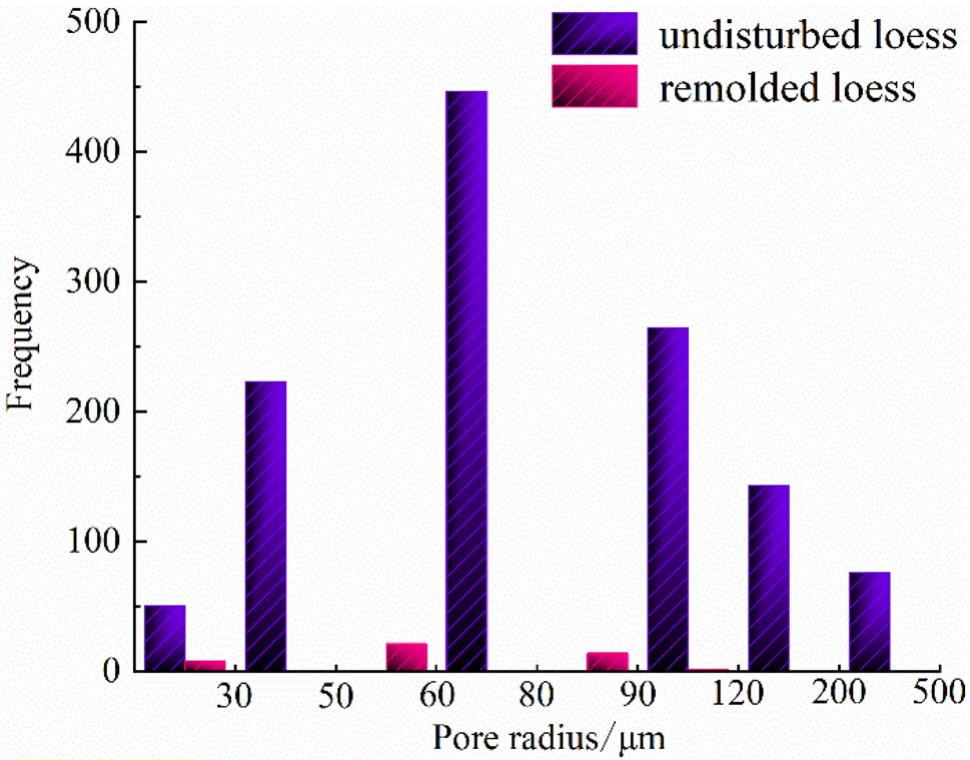
Statistical distribution histogram of specimen pore radius.

**Figure 16:**
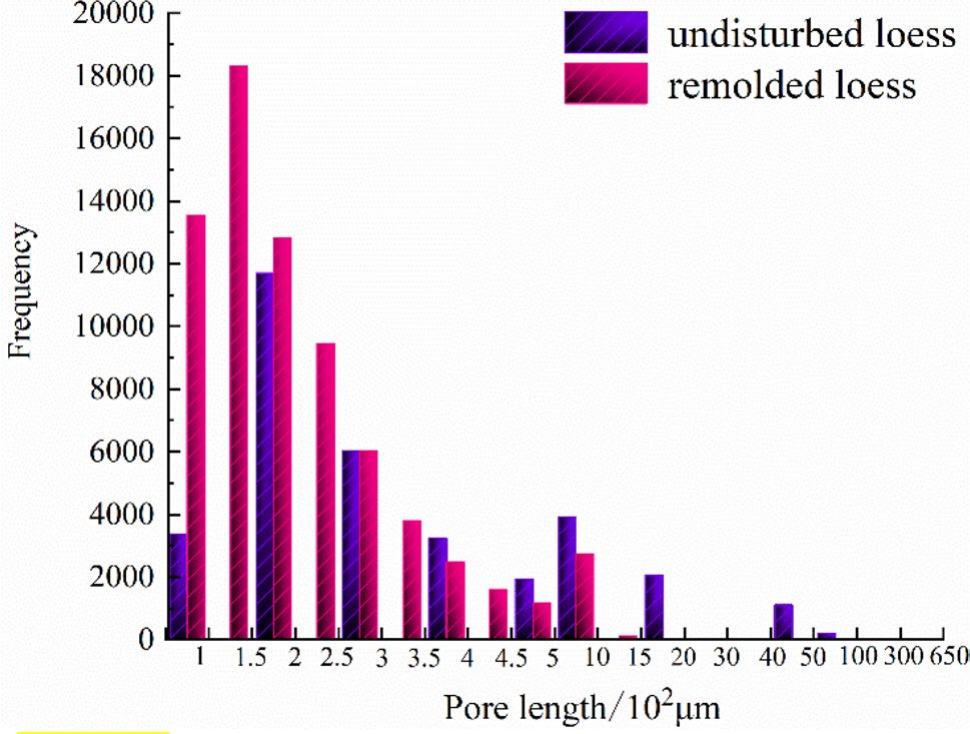
Statistical distribution histogram of specimen pore length.

**Figure 17:**
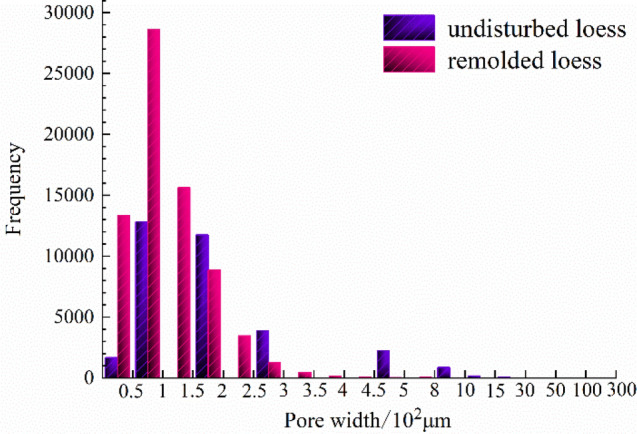
Statistical distribution histogram of specimen pore width.

Figure 15 shows the distribution of the number of each interval of the pore radius of the undisturbed and remolded loess specimens. It can be seen from the figure that the pore size content less than 50 μm in the undisturbed loess specimen is small, accounting for only 22.7% ; when the pore size is greater than 50 μm, the number of pores increases significantly, and the proportion of pore size between 50 and 120 μm can reach 59.1%; the number of pores in the range of 120 μm~200 μm is less than that in the range of 50 ~120 μm, accounting for 11.9% of the total volume; the number of pores larger than 200 μm decreases significantly, accounting for 6.3% of the total volume. The pore size content less than 50 μm in remolded loess specimens is small, accounting for only 16.3%; the number of pores increases obviously when the pore size is larger than 50 μm, and the proportion of pores between 50 μm~120 μm can reach 83.7%. There is no pore with pore size greater than 200 μm in remolded loess. In addition, it can be seen from Figs. 16, 17 that the main distribution range of the length and width of the pores in the undisturbed loess is also larger than that in the remolded loess, and it can also be directly obtained from the displayed three-dimensional pore diagram.

According to the pore type and pore size classification method of Wang^44^, i.e., micro-pores (≤ 50 μm), medium-pores (50−500 μm) and macro-pores (≥ 500 μm), it can be seen that there are pore differences between undisturbed and remolded loess. Specifically, there are a small number of micro-pores, macro-pores and a large number of medium-pores in undisturbed loess specimens, and a small amount of micro-pores and a large number of medium-pores in remolded loess specimens. In addition, combined with the pore characterization parameters of undisturbed and remolded loess provided in Table 6, it can be seen that the average porosity and average pore radius of undisturbed loess are greater than those of remolded loess. It can also be seen that the possibility of large pores in undisturbed loess is great, and the heterogeneity of primary structure is obvious. After artificial compaction, the original structure of loess is gradually destroyed, and the irregular large pores and holes are compressed and deformed under load. The large pores of the support between the loess skeletons are significantly reduced, and the loess specimens tend to be dense in the process of structural remodeling, mainly with medium and micro pores, which is in agreement with the existing research results^45–48^. There are active pores and inert pores in loess pores. When the loess specimen is subjected to external force, the destruction of the microstructure at a certain level will inevitably show the corresponding macroscopic mechanical properties. At this time, the pores existing at the microstructure level are called active pores. The pores that maintain the original state at the microstructure level with little influence on the macroscopic mechanical properties are called inert pores^49^. Therefore, the large pores and a large number of medium pores in undisturbed loess specimens belong to active pores, and a small number of small pores belong to inert pores. In the process of triaxial shear, the stress mainly affects the large pores of the support, and the pore size changes under the stress, which is in agreement with the existing research results^50,51^. This shows that the aerial structure of undisturbed loess plays a key role in the process of shear deformation and failure, and it can also be seen that the different pore characteristics at different microscopic levels make the macroscopic mechanical properties of loess specimens different.

Compared with the previous macroscopic shear test mentioned before, the bracket structure and macropores in undisturbed loess were deformed during shear deformation and failure, but good structural and cementation ensure the strength of undisturbed loess specimens. Due to the parameters such as pore radius, pore volume and shape factor belong to the description of structural characterization, the comprehensive differences of the above structural parameters lead to the difference in the structure of the between undisturbed and remolded loess, which makes the shear strength indexes (cohesion and internal friction angle) different. Among them, the difference of cohesion is large, while the difference of internal friction angle is relatively small. In addition, in the natural state, although there are large pores in the undisturbed loess specimens, there are cements in the skeleton position of the contact between loess particles, which makes the undisturbed loess specimens have high structural stability and strong resistance to external damage. Because the remolded loess is reshaped by manual compaction, the structure of loess particles is destroyed, the particles are rearranged, and the cementing material is also decomposed with the sliding of the skeleton particles and distributed between the particle pores. Therefore, the connection between the loess particles of the remolded loess specimen is relatively loose, and the ability to resist external damage is poor, so that the structural strength of the undisturbed loess is higher than that of the remolded loess.

### Impact of structure on WTER retention characteristics

The previous studies have shown that loess structure affects water retention characteristics^52–54^. When the suction is in the range of 20~80 kPa, with the gradual increase of the suction, the saturation of undisturbed loess is smaller than that of remolded loess; in the range of 80~1000 kPa, the saturation of undisturbed loess is greater than that of remolded loess^55^. Due to the existence of large and medium pores in the undisturbed loess and its own joint structure, water will be discharged rapidly from the loess along pores under low suction. Under high suction, the gas in the pores inhibits the water migration. Because the pores in the remolded loess are uniformly distributed in the fine pores, the gas-induced water migration is not obvious, so that the moisture content of undisturbed loess is higher than that of remolded loess. At the same time, the study shows that the water retention characteristics of loess also have an impact on the strength of loess^20,56–63^. When the moisture content is low, the particles and pores are small, which makes the matrix suction in the pores high. The combined water close to the surface of loess particles is subjected to strong electric molecular gravity, showing strong water film bonding force, and the bonding effect is obvious. The stability of the electric double layer structure on the surface of loess particles is strong, and the loess has large cohesion. Under the condition of high water content, the thickness of the combined water film inside the loess increases, the matric suction decreases, the connection between the water films decreases, the bonding effect decreases, and the cementation between the loess particles gradually loses. The influence of water on the strength of the loess changes from cohesion to lubrication. That is, with the increase of water content, the cementing material between the loess particles is gradually dissolved, resulting in the decrease of the cohesion between the particles, and thus the shear strength of the loess is weakened. Due to the strong structure of undisturbed loess and the cohesion of remolded loess seriously damaged in the process of human disturbance, combined with the macro triaxial shear test, the shear strength of undisturbed loess is greater than that of remolded loess under 50, 100 and 200 kPa suction. This shows that the difference in water retention characteristics between undisturbed and remolded loess specimens is mainly due to the contribution of their own structural differences, which makes the strength of unsaturated loess different.

In this paper, the influence of the micro-structure difference of unsaturated loess on the strength deformation characteristics and mechanical properties of loess is discussed. However, there are some shortcomings in this test. Only the initial micro-structure of undisturbed and remolded loess specimens is analyzed, and the changes of undisturbed and remolded loess specimens in the process of macro-triaxial shear are not presented. Therefore, in the future, the combination of CT machine and triaxial apparatus will be considered to measure the internal structure changes of loess specimens in real time and non-destructively in the process of shear, so as to explain the influence of structural difference on the strength characteristics of loess in more detail. On this basis, the structural evolution equation and structural constitutive model of loess are established, which provides a new idea for studying the nature of loess strength.

## Conclusion

By comparing the structural differences of unsaturated undisturbed and remolded loess under the same physical and mechanical conditions, combined with unsaturated triaxial shear test, scanning electron microscope and CT scanning test, this paper aims to explore the mechanism of structural effect on the mechanical deformation characteristics of loess from the aspects of microstructure and macroscopic mechanics. The main conclusions are as follows:

1. The influence of matric suction on strength parameters of unsaturated undisturbed and remolded loess specimens is different. The cohesion of the two increases linearly with the suction, and the internal friction angle is less affected by the suction. It can be seen that the cohesive force and internal friction angle of undisturbed loess are larger than those of remolded loess, and the difference of internal friction angle is smaller than that of cohesive force. Therefore, under the same suction and confining pressure, the failure stress of intact loess specimens with the same physical properties is greater than that of remolded loess specimens.
2. There are obvious differences in microstructure characteristics between unsaturated undisturbed and remolded loess specimens, mainly in particle morphology and particle contact relationship. The angularity of the undisturbed loess particles is obvious, and the support contact between the particles provides the loess specimen with good stability, and there is a strong cementation between the particles. The shape of the remolded loess particles is close to round shape, the skeleton stability is poor, the contact form between the particles is embedded, and most of the original cemented contact is destroyed. The macroscopic performance is that the original loess can withstand the ultimate destructive force.
3. There are significant differences in the microstructure characteristics of unsaturated undisturbed and remolded loess specimens, mainly in the characterization parameters, such as porosity and pore radius. The average porosity and average radius of undisturbed loess are higher than those of remolded loess, indicating that there are bracket pores in undisturbed loess. In addition, the proportions of pores with pore radius less than 50 μm in undisturbed loess and remolded loess are 22.7% and 16.3%, and the proportions of pores with pore radius from 50 to 120 μm are 59.1% and 83.7%, respectively. In addition, there are pores with pore radius from 120 to 200 μm and pores with pore radius greater than 200 μm in undisturbed loess, and the proportions are 11.9% and 6.3%, respectively. It can be seen that the bracket pores in remolded loess are significantly reduced, but the number of bracket pores in undisturbed loess is small and good structural and cementation ensure the strength of undisturbed loess.
4. The distribution ranges of pore shape factors of undisturbed loess and remolded loess are 0.024~0.050 and 0.022~0.044, respectively. The parameters such as pore radius, shape factor belong to structural parameters, which makes the shear strength indexes different. Among them, the difference of cohesion is large, while the difference of internal friction angle is relatively small. The intact cement between undisturbed loess particles makes provides it with better cohesion than that of remolded loess particles damaged by cement, so that the structural strength of undisturbed loess is higher than that of remolded loess.

The research results provide certain experimental and analysis basis for further revealing the influence mechanism of structure on the mechanical deformation characteristics of unsaturated loess.

## References

[1] Wu Z, Xu S, Chen D (2020). An experimental study of the influence of structural parameters on dynamic characteristics of loess. Soil Dyn. Earthq. Eng..

[2] Jing XY, Zhou WH, Zhu HX (2018). Analysis of soil-structural interface behavior using three-dimensional DEM simulations. Int. J. Numer. Anal. Meth. Geomech..

[3] Tan Q, Tang H, Fan L (2018). In situ triaxial creep test for investigating deformational properties of gravelly sliding zone soil: Example of the Huangtupo 1# landslide, China. Landslides.

[4] Xie Q, Liu J, Han B (2018). Critical hydraulic gradient of internal erosion at the soil-structure interface. Processes.

[5] Zhao M, Chen L, Wang S (2020). Experimental study of the microstructure of loess on its macroscopic geotechnical properties of the Baozhong railway subgrade in Ningxia, China. Bull. Eng. Geol. Env..

[6] Xie X, Li P, Hou X (2020). Microstructure of compacted loess and its influence on the soil-water characteristic curve. Adv. Mater. Sci. Eng..

[7] Jwa B, Ping L, Qi GB (2019). Changes in tensile strength and microstructure of loess due to vibration—ScienceDirect. J. Asian Earth Sci..

[8] Ni WK, Yuan KZ, Lu XF (2020). Comparison and quantitative analysis of microstructure parameters between original loess and remolded loess under different wetting-drying cycles. Sci. Rep..

[9] Jia L, Guo J, Zhou Z (2019). Experimental investigation on strength development of lime stabilized loess. RSC Adv..

[10] Terzaghi K (1943). Theoretical Soil Mechanics.

[11] Casagrande A (1932). The stucture of clay and its importance in foundation engineering. J. Boston Soc. Civ. Eng..

[12] Mitehell JK (1993). Fundamenialsorsoilbehavioy.

[13] Leroudil S, Tavenas F, Brucy F, La Rochelle P, Roy M (1979). Bebavior of destructured natural clay. J. Geotech. Eng. Div. Am. Soc. Civ. Eng..

[14] HadžiNiković GD (2009). The influence of the grain-size distribution and soil structure on the unsaturated shear strength of loess sediments in Belgrade, Central Serbia. Aaales Geologioues De La Peninsule Balkanique.

[15] Matalucci RV, Abdel-Hady M, Shelton JW (1970). Influence of microstructure of loess on triaxial shear strength. Eng. Geol..

[16] Matalucci RV, Abdel-Hady M, Shelton JW (1970). Influence of grain orientation on direct shear strength of a loessial soil. Eng. Geol..

[17] Matalucci, R. V. *The Microstructure of Loess and Its Relationship to Engineering Properties. Microstructure of Loess & Its Relationship to Engineering Properties* (1970).

[18] Li YR (2018). A review of shear and tensile strengths of the Malan Loess in China. Eng. Geol..

[19] Wen BP, Yan YJ (2014). Influence of structure on shear characteristics of the unsaturated loess in Lanzhou, China. Eng. Geol..

[20] Gao B, Su L (2020). Triaxial mechanical testing of undisturbed unsaturated loess. Soil Mech. Found. Eng..

[21] Haeri SM, Khosravi A, Garakani AA (2017). Effect of soil structure and disturbance on hydromechanical behavior of collapsible loessial soils. Int. J. Geomech..

[22] Xu L, Lan TG, Mu QY (2021). Effects of structure on the compression behavior of unsaturated loess. Int. J. Geomech..

[23] Yao ZH, Chen ZH, Fang XW, Li W, Su LH (2010). Elastoplastic damage seepage—consolidation coupled model of unsaturated undisturbed loess and its application. Acta Geotech..

[24] Shi B (1996). Review and prospect of microstructure research of cohesive soil. J. Eng. Geol..

[25] Samoilych KO (2016). Determination of influence of the microstructure on the physical and mechanical properties of loess soils in Dnieper region. J. Geol. Geogr. Geoecol..

[26] Sadeghi J, Zhou B (2020). Effect of microstructure on shear strength and dilatancy of unsaturated loess at high suctions. Can. Geotech. J..

[27] Wang Y, Yang H, Jing X (2021). Structural characteristics of natural loess in Northwest China and its effect on shear behavior. Geotech. Geol. Eng..

[28] Zhang JW, Mu QY, Garg A (2020). Shear behavior of unsaturated intact and compacted loess: A comparison study. Environ. Earth Sci..

[29] Zhang Y, Hu Z, Li L (2018). Improving the structure and mechanical properties of loess by acid solutions—An experimental study. Eng. Geol..

[30] Meng J, Li XA (2019). Effects of carbonate on the structure and properties of loess and the corresponding mechanism: An experimental study of the Malan loess, Xi'an area, China. Bull. Eng. Geol. Env..

[31] Hu W, Liu H, Wang T (2021). Study on the effect of resistivity and meso-structure evaluation of alkali solution strengthening loess: A case study. IOP Conf. Ser. Earth Environ. Sci..

[32] Wei YN, Fan W, Yu B, Deng LS, Wei T (2020). Characterization and evolution of three-dimensional microstructure of Malan loess. Catena.

[33] Ye W, Bai Y, Cui C (2020). Deterioration of the internal structure of loess under dry-wet cycles. Adv. Civ. Eng..

[34] Chen ZH, Guo N (2019). New developments of mechanics and application for unsaturated soils and special soils. Rock Soil Mech..

[35] Jiang MJ, Sun RH, Li T, Liu J (2019). A three-dimensional cementation contact model for unsaturated structural loess. Chin. J. Geotech. Eng..

[36] Fredlund DG, Morgenstern NR, Widger RA (1978). The shear strength of unsaturated soils. Can. Geotech. J..

[37] Wei YZ, Yao ZH, Chong XL (2021). Microstructural characteristics of unsaturated Q3 loess and its influence mechanisms on strength properties. Chin. J. Geotech. Eng..

[38] Deng L, Fan W, Liu S, Chang Y, Dai Y, Li Y (2020). Quantitative research and characterization of the loess microstructure in the Bai Lu Tableland, Shaanxi Province, China. Adv. Civ. Eng..

[39] Fukushima Y, Higo Y, Matsushima T (2021). Liquid bridge contribution to shear behavior of unsaturated soil: Modeling and application to a micromechanics model. Acta Geotech..

[40] Kim H, Park SW (2020). DEM simulation for shear behavior in unsaturated granular materials at low-stress state. Comput. Geotech..

[41] Zhang X, Lu Y, Li X (2019). Microscopic structure changes of Malan loess after humidification in South Jingyang Plateau, China. Environ. Earth Sci..

[42] Xu JB, Cao BH, Yu YL, Luo YZ (2021). Sensitivity analysis of meso-parameters in loess triaxial test based on PFC^3D^. J. Eng. Geol..

[43] Jing X, Xie WL, Shan S (2021). Discrete element simulation study on micromechanical characteristics of undisturbed and remolded loess in biaxial test. Bull. Geol. Sci. Technol..

[44] Wang YY, Teng ZH, Yue LP (1982). The surface structure of quartz particles in loess and the genesis of Chinese loess. Acta Geogr. Sin..

[45] Wang JD, Li P, Ma Y, Vanapalli SK (2019). Evolution of pore-size distribution of intact loess and remolded loess due to consolidation. J. Soils Sediment..

[46] Nan J, Peng J, Zhu F, Ma P, Liu R, Leng Y, Meng Z (2021). Shear behavior and microstructural variation in loess from the Yan'an area, China. Eng. Geol..

[47] Li Y, He S, Deng X (2018). Characterization of macropore structure of Malan loess in NW China based on 3D pipe models constructed by using computed tomography technology. J. Asian Earth Sci..

[48] Xu P, Zhang Q, Qian H (2021). An investigation into the relationship between saturated permeability and microstructure of remolded loess: A case study from Chinese Loess Plateau. Geoderma.

[49] Tan YZ, Kong LW, Guo AG, Wan Z (2010). Research on effect of compaction on pore size distribution of laterite soil. Rock Soil Mech..

[50] Xie X, Qi S, Zhao F (2017). Creep behavior and the microstructural evolution of loess-like soil from Xi'an area, China. Eng. Geol..

[51] Li ZQ, Qi ZY, Qi SW (2021). Microstructural changes and micro-macro‑relationships of an intact, compacted and remolded loess for land‑creation project from the Loess Plateau. Environ. Earth Sci..

[52] Ng C, Sadeghi H, Hossen SB (2016). Water retention and volumetric characteristics of intact and re-compacted loess. Can. Geotech. J..

[53] Li X, Hu C, Li F, Gao H (2020). Determining soil water characteristic curve of lime treated loess using multiscale structure fractal characteristic. Sci. Rep..

[54] Li L, Li XA, Wang L (2020). The effects of soil shrinkage during centrifuge tests on SWCC and soil microstructure measurements. Bull. Eng. Geol. Env..

[55] Wei F, Yao ZH, Su LH (2015). Study on water holding capacity of unsaturated undisturbed and remolded loess of Q3. Geotech. Invest. Surv..

[56] Xu X, Li Q, Lai Y (2019). Effect of moisture content on mechanical and damage behavior of frozen loess under triaxial condition along with different confining pressures. Cold Reg. Sci. Technol..

[57] Tian, J., Yao, W., Xuan, Z., *et al*. Experimental study on the effect of moisture content and the freeze-thaw cycle on unsaturated loess strength damage. In *International Conference on Smart City & Systems Engineering. *(IEEE, 2017).

[58] Vaughan PR, Skinner AE, Lupini JF (1981). The drained residual strength of cohesive soils. Geotechnique.

[59] Wang X, Wang J, Zhan H (2019). Moisture content effect on the creep behavior of loess for the catastrophic Baqiao landslide. Catena.

[60] Yan CG, Qi W, Yu X (2018). Experimental study of barrier effect on moisture movement and mechanical behaviors of loess soil. Eng. Geol..

[61] Zuo C, Liu D, Ding S (2016). Micro-characteristics of strength reduction of tuff residual soil with different moisture. KSCE J. Civ. Eng..

[62] Liu JW, Fan HH, Song XY (2020). Characteristics of shear strength and deformation of compacted Q3 loess. Soil Mech. Found. Eng..

[63] Hao YZ, Wang TH, Wang JJ (2019). Structural properties of unsaturated compacted loess for various sample moisture contents. Arab. J. Geosci..

